# Substitution of histidine 95 by tyrosine in the prion protein causes spontaneous neurodegeneration in transgenic mice

**DOI:** 10.1371/journal.ppat.1013554

**Published:** 2025-10-16

**Authors:** Juan-María Torres, Alba Marín-Moreno, Juan-Carlos Espinosa, Sara Canoyra, Anna Burato, Arianna Ciullini, Chiara Maria Giulia De Luca, Edoardo Bistaffa, Fabio Moda, Giuseppe Legname

**Affiliations:** 1 Centro de Investigación en Sanidad Animal (CISA-INIA), CSIC: Consejo Superior de Investigaciones Cientificas, Valdeolmos, Madrid, Spain; 2 Departamento de Bioquímica y Biología Molecular, Facultad de Ciencias Químicas, Universidad Complutense de Madrid UCM, Madrid, Spain; 3 Laboratory of Prion Biology, Department of Neuroscience, Scuola Internazionale Superiore di Studi Avanzati (SISSA), Trieste, Italy; 4 Unit of Laboratory of Medicine – Laboratory of Clinical Pathology, Fondazione IRCCS Istituto Neurologico Carlo Besta, Milan, Italy; 5 Division of Neurology 5 and Neuropathology, Fondazione IRCCS Istituto Neurologico Carlo Besta, Milan, Italy; 6 Department of Medical Biotechnology and Translational Medicine, University of Milan, Milan, Italy; 7 ELETTRA Sincrotrone Trieste S.C.p.A, Basovizza, Trieste, Italy; National Institutes of Health, NIAID, UNITED STATES OF AMERICA

## Abstract

Prion diseases are neurodegenerative disorders caused by a change in conformation of the prion protein from the cellular form (PrP^C^) to a misfolded isoform (PrP^Sc^). PrP^C^ is a copper binding protein via histidine residues in the octapeptide repeats (OR) and the non-OR region located at the N-terminus. Although the functional implication of copper binding to PrP^C^ is still under investigation, copper may play a role in prion disease. In this study, we describe transgenic mice expressing mouse prion protein replacing histidine 95 by tyrosine (PrP H95Y) to disrupt the non-OR copper-binding site. Transgenic mice overexpressing PrP H95Y showed clinical signs and died at about 100 days with spongiform degeneration and PK-resistant PrP. Inoculation of brain homogenate from mice overexpressing PrP H95Y to *Tga20* mice expressing wild-type PrP also causes lethal, spongiform encephalopathy. We conclude that this substitution could promote PrP^C^-PrP^Sc^ conversion and induce spontaneous prion disease *in vivo*.

## Author summary

Prion diseases are neurodegenerative disorders characterized by the misfolding of the normal cellular prion protein (PrP^C^) into a pathogenic isoform (PrP^Sc^). PrP^C^ binds copper through histidine residues located in the N-terminal domain, and copper binding has been proposed to influence PrP^C^ structure and its conversion to PrP^Sc^. In this study, we aimed at disrupting this copper coordination by generating transgenic mice expressing a mutant prion protein, in which histidine 95 was replaced by tyrosine (PrP H95Y). Mice overexpressing PrP H95Y developed spontaneous prion disease with typical clinical signs, spongiform degeneration, and protease-resistant PrP accumulation. Furthermore, brain homogenates from these mice were able to transmit the disease to mice expressing wild-type PrP, validating the infectious nature of the spontaneously generated prions. These results demonstrate that interfering with copper coordination at H95 is sufficient to promote prion conversion *in vivo*, further highlighting the connection between copper binding and prion pathogenesis.

## Introduction

Prion diseases, in human and animals, are a group of rare and fatal neurodegenerative diseases [1]. They have a common feature in the aberrant metabolism of the prion protein (PrP). During the pathogenesis of these diseases, the cellular isoform of the prion protein (PrP^C^) adopts a pathogenic misfolded conformation denoted as prion or PrP^Sc^, which accumulates in the central nervous system (CNS) and causes neurodegenerative diseases [2]. The normal, cellular form, PrP^C^, can be divided in two regions, a flexible unstructured N-terminal region and a C-terminal globular region. Based on NMR spectroscopy and X-ray crystallography studies, C-terminal PrP^C^ domain (residues 127–230) possesses folded structure with a predominantly α-helices and small β-sheet content [3]. The N-terminal region (residues 23–126) does not adopt any detectable folding and for this reason this moiety can be considered as a partially intrinsic disordered protein. This N-terminal region has a unique primary sequence since it contains highly conserved octapeptide repeats (OR) with a distinctive consensus sequence, PHGGGWGQ [4]. This region contains up to four OR sequences in humans and mice and each OR is able to coordinate a copper ion with high affinity through its histidine (H) residue, although alternative geometries may exist [5]. Two additional histidine residues able to bind copper are found in the 90–111 region, namely at position 95 and 110 in mouse PrP [6]. This additional region is denoted as the non-OR region [4]. Several studies have evaluated the role of copper in the conversion propensity of PrP^C^ to PrP^Sc^ with controversial results [7,8]. Structural studies have underlined the importance of the non-OR region and, in particular, histidines 95 and 110 [9]. A mutagenesis study evaluated the susceptibility to prion inoculation in transgenic mice where each histidine at the N-terminus was substituted with glycine. While the concomitant substitution of all histidines at the OR did prolong the incubation time upon inoculation with prions, a single substitution at position 95 accelerated the disease [10]. In addition, structural studies have shown that the refolding of the N-terminal moiety strictly depends on copper coordination [11]. In fact, changing the histidine in position 95 with a tyrosine causes the protein to acquire a conformation that could be more susceptible to prion replication, implying for this region a role as molecular switch in rendering the protein more prone to prion conversion [9,12].

In this study, we evaluated the effect of histidine substitution at position 95 with a tyrosine *in vivo*. By generating several transgenic mouse lines bearing this substitution, we were able to analyze in detail the outcome in terms of prion conversion, replication and disease.

## Materials and methods

### Ethics statement

We carried out animal experiments in strict accordance with the recommendations present in the guidelines of the Code for Methods and Welfare Considerations in Behavioral Research with Animals (Directive 2010/63/ EU). All efforts were made to minimize suffering. Experiments were approved by the Committee on the Ethics of Animal Experiments of the Instituto Nacional de Investigación y Tecnología Agraria y Alimentaria (Madrid, Spain; Permit numbers: PROEX 018/17, PROEX 030/18 and PROEX 97.5/23).

### Plasmids construction

#### Primer design.

Primers were designed using the Primer Blast (NCBI) and QuikChange Primer Design (Agilent Technologies) (See Tables 1 and 2).

**Table 1:** Primers for His/Tyr mutations.

Primer	Sequences
H60Y-F	CAGCCCTACGGTGGTGGCTGGGGACAA
H60Y-R	TTGTCCCCAGCCACCACCGTAGGGCTG
H68Y-F	GGGGACAACCCTATGGGGGCAGCTGG
H68Y-R	CCAGCTGCCCCCATAGGGTTGTCCCC
H76Y-F	AGCTGGGGACAACCTTATGGTGGTAGTTGGG
H76Y-R	CCCAACTACCACCATAAGGTTGTCCCCAGCT
H84Y-F	TGGGGTCAGCCCTATGGCGGTGGATGG
H84Y-R	CCATCCACCGCCATAGGGCTGACCCCA
H95Y-F	CAAGGAGGGGGTACCTATAATCAGTGGAACAAGC
H95Y-R	GCTTGTTCCACTGATTATAGGTACCCCCTCCTTG
H110Y-F	AAAAACCAACCTCAAGTATGTGGCAGGGGCTGC
H110Y-R	GCAGCCCCTGCCACATACTTGAGGTTGGTTTTT

**Table 2:** Primers for H95X mutations.

Primer	Sequences
H95R-F	CAAGGAGGGGGTACCAGGAATCAGTGGAACAAGC
H95R-R	GCTTGTTCCACTGATTCCTGGTACCCCCTCCTTG
H95KF	CAAGGAGGGGGTACCAAGAATCAGTGGAACAAGC
H95K-R	GCTTGTTCCACTGATTCTTGGTACCCCCTCCTTG
H95D-F	CAAGGAGGGGGTACCGACAATCAGTGGAACAAGC
H95D-R	GCTTGTTCCACTGATTGTCGGTACCCCCTCCTTG
H95E-F	CAAGGAGGGGGTACCGAGAATCAGTGGAACAAGC
H95E-R	GCTTGTTCCACTGATTCTCGGTACCCCCTCCTTG
H95S-F	CAAGGAGGGGGTACCAGCAATCAGTGGAACAAGC
H95S-R	GCTTGTTCCACTGATTGCTGGTACCCCCTCCTTG
H95T-F	CAAGGAGGGGGTACCACCAATCAGTGGAACAAGC
H95T-R	GCTTGTTCCACTGATTGGTGGTACCCCCTCCTTG
H95N-F	CAAGGAGGGGGTACCAACAATCAGTGGAACAAGC
H95N-R	GCTTGTTCCACTGATTGTTGGTACCCCCTCCTTG
H95Q-F	CAAGGAGGGGGTACCCAGAATCAGTGGAACAAGC
H95Q-R	GCTTGTTCCACTGATTCTGGGTACCCCCTCCTTG
H95C-F	CAAGGAGGGGGTACCTGCAATCAGTGGAACAAGC
H95C-R	GCTTGTTCCACTGATTGCAGGTACCCCCTCCTTG
H95G-F	CAAAAACCAACATGAAGGCTATGGCAGGGGCTGCGG
H95G-R	CCGCAGCCCCTGCCATAGCCTTCATGTTGGTTTTTG
H95P-F	CAAGGAGGGGGTACCCCTAATCAGTGGAACAAGC
H95P-R	GCTTGTTCCACTGATTAGGGGTACCCCCTCCTTG
H95A-F	CAAGGAGGGGGTACCGCCAATCAGTGGAACAAGC
H95A-R	GCTTGTTCCACTGATTGGCGGTACCCCCTCCTTG
H95V-F	CAAGGAGGGGGTACCGTGAATCAGTGGAACAAGC
H95V-R	GCTTGTTCCACTGATTCACGGTACCCCCTCCTTG
H95I-F	CAAGGAGGGGGTACCATCAATCAGTGGAACAAGC
H95I-R	GCTTGTTCCACTGATTGATGGTACCCCCTCCTTG
H95L-F	CAAGGAGGGGGTACCCTGAATCAGTGGAACAAGC
H95L-R	GCTTGTTCCACTGATTCAGGGTACCCCCTCCTTG
H95M-F	CAAGGAGGGGGTACCATGAATCAGTGGAACAAGC
H95M-R	GCTTGTTCCACTGATTCATGGTACCCCCTCCTTG
H95F-F	CAAGGAGGGGGTACCTTCAATCAGTGGAACAAGC
H95F-R	GCTTGTTCCACTGATTGAAGGTACCCCCTCCTTG
H95W-F	CAAGGAGGGGGTACCTGGAATCAGTGGAACAAGC
H95W-R	GCTTGTTCCACTGATTCCAGGTACCCCCTCCTTG

#### Cloning of MoPrP variants plasmid for cell transfection.

The mutations were inserted into pcDNA-MoPrP or pcDNA-MoPrP3F4 using the QuikChange mutagenesis kit (Agilent Technologies) according to manufacturer’s instructions.

PCR parameters were as follows: melting temperature: 95°C for 30 sec; annealing temperature: 55°C for 45 sec; extension temperature: 68°C for 5 min. These three steps were cycled 16 times. Strand extension was completed at 72°C for 5 min and reactions cooled to 4°C. 10 ng of template DNA and 2.5U *PhuUltra* DNA polymerase was used in each reaction. PCR products were digested by DpnI (1 μL per 20 μL PCR reaction for 1hour at 37°C) prior to transformation into XL-1 Blue supercompetent cells.

Plasmid DNA was purified using the QIAprep Miniprep Kit (Qiagen). All the constructs were verified by sequencing.

#### Cloning of full-length MoPrP variants plasmid for Tg mice construction.

The MoPrP(1–254, H95Y) was amplified from plasmid pcDNA-MoPrP-H95Y by PCR using these primer:

- 5’Bsi-MoPrP: 5’-CCT AGT GGT ACC TCG TAC GCA GTC ATC ATG GCG AAC CTT GGC TAC TGG-3’
- 3’Fse-MoPrP: 5’-CGC TCA CAA TCG CGG CCG GCC TCA TCC CAC GAT CAG GAA GAT GAG G-3’

The PCR products were then inserted into pJB1 (a modified vector from MoPrP.Xho vector [13], in which BsiWI and FseI sites replaced the XhoI site) using BsiWI and FseI restriction sites.

The PCR were performed using the Phusion DNA polymerase (New England Biolab) according to manufacturer’s instructions. Plasmid DNA was purified using the QIAprep Miniprep Kit (Qiagen). All the constructs were verified by sequencing.

The pJB1 were kindly provided by the group of Dr. Glenn Telling (Colorado State University, USA).

### Generation and characterization of transgenic mice

MoPrP H95Y transgenes containing mouse regulatory sequences were excised from the plasmid pJB::MoPrP(1–254, H95Y) using the restriction endonuclease NotI. These fragments were purified and then microinjected into the pronucleus of 200 fertilized FVB mice eggs, which were implanted into pseudopregnant FVB females. This work was carried out at the transgenic mouse facility of Cyagen Biosciences Inc (USA).

For founder’s identification, DNAs were extracted from tail biopsies of founders mice by use of an Extract-N-Amp tissue PCR kit (Sigma-Aldrich) following the manufacturer’s instructions. The presence of the transgene in these founders was identified by PCR amplification using specific primers for mouse PrP exon 2 and the mouse mutated PrP ORF. The primers used were 5’-GAACTGAACCATTTCAACCGAG-3 and 5 = ’-AGAGCTACAGGTGGATAACC-3’. MoPrP^+/+^ MoPrP H95Y^+/-^ founders (identified by the letter “x”) were backcrossed with homozygous PrP null animals (Mo founders’PrP^-/-^) to obtain mice knock-out for the wild-type (wt) murine allele (MoPrP^-/-^ MoPrP H95Y^+/-^, identified by the letter “k”). The absence of the endogenous wt murine PrP ORF in the transgenic mice thus generated was confirmed by PCR amplification using the primers 5’-TAGATGTCAAGGACCTTCAGCC-3’ and 5’-GTTCCACTGATTATGGGTACC-3’.

To determine PrP^C^ expression levels in the new generated mouse lines, whole brains were homogenized in extraction buffer (0.5% NP-40, 1% sodium deoxycholate, 10 mM EDTA in phosphate-buffered saline (PBS), pH 7.4, with Complete protease inhibitor cocktail (Roche). Samples were precleared by centrifugation at 2,000 x g for 5 min, after which an equal volume of 2X SDS reducing sample loading buffer was added to all samples, and each one was boiled for 5 min before being loaded onto an SDS-12% polyacrylamide gel. For immunoblotting experiments, the monoclonal antibody (MAb) 12B2 [14] was used at a concentration of 1 µg/mL. 12B2 recognizes the 88WGQGG92 epitope of the mouse PrP sequence. Immunocomplexes were detected using horseradish peroxidase-conjugated anti-mouse IgG. Immunoblots were developed with enhanced chemiluminescence.

Finally, a further characterization of the spontaneous disease developed by the transgenic mice was done. Neurological status was assessed twice a week and the appearance of clinical signs were carefully recorded. Mean survival times and attack rates were calculated.

### Cell culture

N2a and ScN2a cell lines were cultured in Opti-MEM (GIBCO) media supplemented with 10% fetal bovine serum (FBS) and 1% penicillin-streptomycin, and incubated at 37°C, 5% CO_2_. Transfections with RML strain were performed using Effectene Transfection Reagent (Qiagen) according to the manufacturer’s guidelines. For stable transfections, 48 hours after transfection cells were split into fresh medium containing 1mg/mL Geneticin (Invitrogen). The selective medium was changed every 3–4 days until Geneticin-resistant foci could be identified. After that, the stable cell lines were maintained in medium containing 400 µg/mL Geneticin.

### Analysis of proteinase K-resistant PrP (PrP^res^) by western blot (WB) in cell lysates

The protocol used is extensively reported in Giachin et al. (2015) [12], and precisely followed as already proved to be effective in our laboratory.

### Biochemical assays

For glycosidase assay, 9 μL cell lysate or brain homogenates (1–20 µg protein) were denatured with 1 X glycoprotein denaturing buffer (0.5% sodium dodecylsulfate (SDS), 1% β-mercaptoethanol) at 95°C for 5 minutes. After that, samples were chilled on ice and centrifuged 10 seconds before incubation with 1500 U of Endo-H or PNGase-F in 1 X G5 or G7 reaction buffer, 1% NP-40 at 37°C overnight with vigorous shaking. The reaction was stopped by freezing at -20°C.

For detergent insolubility assay, postnuclear supernatants were prepared from cell lysate or brain homogenates by centrifugation for 5 min at 900 x g. Postnuclear supernatants containing 50 μg of total protein were adjusted to a final volume of 0.5 mL in lysis buffer containing Complete Protease Inhibitors (Roche). Samples were incubated on ice for 20 min before being centrifuged at 100,000 x g, 1 hour, 4°C. The supernatant was recovered and the pellet resuspended in an equal volume of 5% (w/vol) Sarkosyl buffered with 10mM Tris, pH 8.0. Samples were then methanol precipitated for further analyses by Western blot (WB).

### Histological analysis

Brains were fixed in Carnoy solution [15], then dehydrated in graded ethanol solutions, cleared in xylene, and embedded in paraffin. Seven-micrometer sagittal sections were obtained by using a sliding microtome and mounted on polylysine-coated slides. Dewaxed sections were stained with hematoxylin-eosin (H&E). Spongiform profiles were determined on H&E-stained sections, by scoring the vacuolar changes in nine standard gray matter areas as described [16].

### Analysis of proteinase K-resistant PrP (PrP^res^) by western blot (WB) in brain tissue

A total of 175 mg of brain tissue was homogenized in 5% glucose in distilled water in grinding tubes (Bio-Rad) and adjusted to 10% (w/vol) by using a TeSeE Precess 48 homogenizer (Bio-Rad) following the manufacturer’s instructions. To determine the presence of PrP^res^ in transgenic mouse brains, 100 µL of 10% brain homogenate were analyzed by WB as previously described [4]. Briefly, 100 µL of a 10% (w/v) brain homogenate were diluted in a 10% (w/v) negative sheep brain homogenate, to obtain a 200 µL final volume. Homogenates were incubated for 10 min at 60°C with 200 µL of an 80 µg/mL proteinase K (PK, Roche) solution. PrP^res^ was recovered as a pellet after addition of 200 µL of buthanol and a centrifugation at 15,000 x g for 7 min at 20°C. Supernatants were discarded and pellets were dried inverted over absorbent paper for 5 min. Pellets were solubilised in Laemmli buffer and samples were incubated for 5 min at room temperature, and heated at 100°C for 5 min. Samples were centrifuged at 20,000 x g for 15 min at 20°C and supernatants were recovered and loaded on a 12% Bis-Tris Gel (Criterion XT, BioRad or NuPage, Invitrogen). Proteins were electrophoretically transferred onto PVDF or nitrocellulose membranes (Millipore). Membranes were blocked O/N with 2% BSA (Sigma) blocking buffer. For immunoblotting, membranes were incubated with the 12B2 [17] which recognizes the 88WGQGG972 epitope of the mouse PrP sequence.

Immunocomplexes were detected with horseradish peroxidase-conjugated anti-mouse IgG (Amersham Pharmacia Biotech) after incubating the membranes for 1 hour, and immunoreactivity was visualized by chemiluminescence with ECL Plus (GE Healthcare Amersham Biosciences). Images were captured using ChemiDoc XRS+System and Image Lab 5.2.1 Software was used for images processing and densitometry analysis. Values were normalised against β-actin levels.

PrP^res^ deglycosylation was performed after PK treatment by using a peptide-N-glycosidase (PNGaseF+) F kit (New England Biolabs) according to the manufacturer’s instructions.

### Solubility assay

The analysis of the insoluble fraction of the PrP from transgenic mice was performed by differential centrifugation following the protocol established by Pririsinu and colleagues [18]. As a modification of the published protocol, the 10% brain homogenates from transgenic mice were solubilized in 100mM TrisHCl, pH 7.4 (to a 6% brain homogenate) and separation of PrP^Sc^ and PrP^C^ was based on a centrifuge in the presence of 5% sarcosyl.

### Transmission studies

To characterize the transmissibility of the spontaneous disease developed in the MoPrP H95Y transgenic mice, k907, *Tga20* and FVB WT mice were intracranially challenged with x905, k905 and x907 inocula (see Table 5). *Tga20* and FVB WT mice were also challenged with FVB WT brains inoculated with K905 inoculum (second passage). Inocula were prepared from diseased brain tissues as 10% (w/vol) homogenates in 5% glucose.

Groups of 6–9 individual identified animals (6–7 weeks old) were anesthetized and inoculated intracerebrally with 20 µL of 10% brain homogenate in the right parietal lobe, using a 25-gauge disposable hypodermic needle. As a negative control, groups of animals from each line were left un-inoculated. Mice were observed daily and their neurological status was assessed twice a week. When the progression of the disease was evident, or at the end of their life span (>600 days), mice were euthanized for ethical reasons. During necropsy, the brain was harvested at -20°C for determination of the presence of PrP^res^ by WB. Survival time was expressed as the mean number of survival days postinoculation (dpi) for all the PrP^res^-positive mice, with the standard error included. Attack rate was determined as the proportion of PrP^res^-positive mice among all the inoculated mice.

### Immunohistochemistry

Seven-μm thick serial sections were immunostained with monoclonal antibodies to PrP (see Table 3 for details) and polyclonal antibodies to astrocyte activation (GFAP, 1:2000; Dako). Before PrP immunostaining, the sections were sequentially treated with different concentrations of proteinase K (20 μg/mL, 10 μg/mL; 7,5 μg/mL; 5 μg/mL; 2,5 μg/mL for 5 min at room temperature) and guanidine isothiocyanate (3M, 20 min), and non-specific binding was prevented using ARK kit (Dako). Immunoreactions were visualized using 3–3′-diaminobenzidine (DAB, Dako) as a chromogen.

**Table 3:** List of anti-PrP antibodies used in the this study.

Clone	PrP epitope	Reference
EB8	26-34	Kosmac et al., 2011 [19]
W226	145-153	Petsch et al., 2011 [20]
Saf84	166-172	Perrier et al., 2004 [21]
*12B2*	89-93	Langeveld et al., 2006 [14]
3F4	109–112	Kascsak et al., 1987 [22]
Sha31	144-151	Féraudet et al., 2005 [23]
Saf32	octarepeat	Féraudet et al., 2005 [23]
Saf-61	142-160	Demart et al., 1999 [24]
6H4	144-152	Krejciova et al., 2017 [25]

### Statistical analysis

Data were expressed as mean plus standard error of mean (SEM; sd√n). Data were compared by 2-tailed t-tests and considered significantly different when P < 0.05. Degree of significance was expressed as follows: P < 0.05*; P < 0.01**; P < 0.001***, unless otherwise specified.

## Results

### The non-OR MoPrP H95Y mutation promotes prion conversion

**Fig 1:**
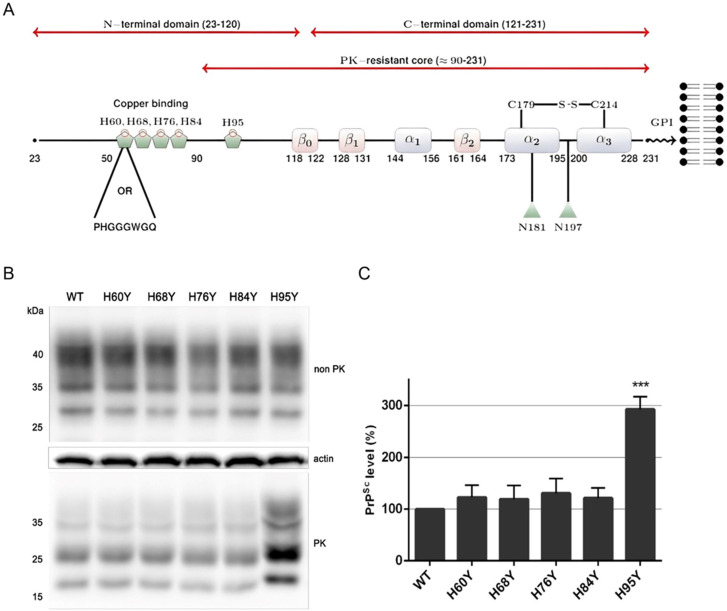
A) Schematic representation of PrP and the histidine residues involved in this study. The N-terminal domain contains four octapeptide repeats and one non-octarepeat site, each characterized by high affinity for copper ions (Cu 2+). The histidine residues involved in copper coordination are shown as pentagons. The globular domain contains three α-helixes and three short β-strands. The disulphide bond and the two glycosylation sites are shown at the C-terminal domain. GPI represents the glycosylphosphatidylinositol anchor; B) Immunoblot showing the expression of either WT, H60Y, H68Y, H76Y, H84Y or H95Y tagged with a 3F4 epitope in stably transfected N2A cells, and detected using an anti-3F4 antibody C) PrP^Sc^ level comparison in each individual mutant. Tyrosine in exchange of histidine does not change the overall PrP expression level in both octarepeat and non-octarepeat regions, but only the non-OR mutant resulted in higher PrP^Sc^ levels compared to both wt PrP and OR mutants. Experiments were replicated three times.

Expression of exogenous PrP in a prion-infected mouse neuroblastoma (ScN2a) cell line results in its conversion to proteinase-K resistant PrP (PrP^res^), a useful tool to study prion conversion. In this study, we engineered the MoPrP constructs to include the human-specific 3F4 epitope (Met substitutions at residues 108 and 111), known not to interfere with the conversion process (S1 Fig) and to allow the discrimination of exogenous MoPrP from endogenous MoPrP [22,26,27]. To investigate the role of copper binding sites of prion protein, we previously created MoPrP mutants in which single histidine residues along the entire N-terminal domain (Fig 1A) were replaced by tyrosine (denoted as H60Y, H68Y, H76Y, H84Y and H95Y mutations).

The substitutions of histidine by tyrosine in MoPrP constructs were not toxic to cells (S2 Fig) and did not affect PrP expression with all constructs sharing a similar PrP expression level (Fig 1B, upper panel). Meanwhile, the PK-resistant profiles displayed interesting results. When histidine in OR was replaced by tyrosine, it had no significant effect to prion conversion, and all OR mutants (H60Y, H68Y, H76Y, H84Y) displayed the same PK-resistant PrP^Sc^ levels as wt PrP. Conversely, the non-OR mutant (H95Y) yielded a significantly higher PrP^Sc^, around 3 times more than the others (Fig 1B, lower panel; 1C), providing evidence for the role of this mutation in prion replication, as previously reported [12].

Next, we wondered whether the large increment in PrP^res^ in MoPrP H95Y expressed in ScN2a cells was due to aspecific change in that amino acid or might underline a biochemical change in PrP. In other words, we wanted to assess if the increase in PrP^res^ was due to the lack of H95 or to the introduction of the tyrosine residue. We therefore changed H95 with every single amino acid and estimated the change in PrP^res^.

In order to further investigate the potential role of the fifth copper-binding site in prion conversion, we created the amino acid scanning at H95, in which all possible amino acid substitutions were introduced. Then, the influence of this substitution on the conversion efficiency was evaluated based on PK-digestion assay in ScN2a cells.

**Fig 2:**
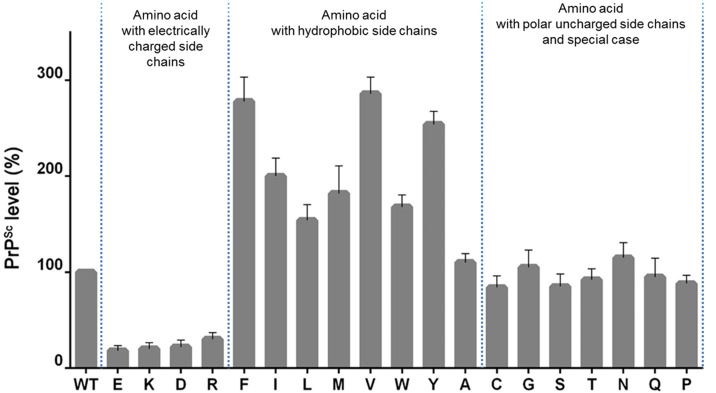
Effects of amino acid substitutions at His95 on prion conversion. Hydrophobic residues specifically enhance the conversion of the prion protein into a proteinase K (PK) resistant form, while amino acids with electrically charged side chains seem to have the opposite effect. This confirms His95 as a “hot spot” for prion conversion. Experiments were replicated three times.

WB analysis of ScN2a cells transfected with H95X mutants revealed the following results (Fig 2). The expression levels of H95X mutants were equal to those of wt PrP, but the PK-resistant level showed large variation. When H95 was replaced by a neutral or small amino acid (A, G, C, S, T, N, Q, P), the PrP^res^ level was equal to wt PrP. In case of hydrophobic amino acids (F, Y, L, M, V, W, I), the prion conversion was largely enhanced. A high level of PrP^res^ was found in ScN2a cell expressing these mutants, and in some specific cases, such as F, V, I, the PrP^res^ level was 2–3 times more than wt PrP. Meanwhile, only a small amount of PrP^res^ was detected in the mutants with charged amino acid residues at H95, suggesting that prion replication may be inhibited in these mutants. These data showed the hydrophobicity of amino acid at H95 might have an impact on PrP^C^-PrP^Sc^ conversion. They also revealed the fifth copper-binding site (that is H95) as a “hot spot” for prion conversion, because by changing the amino acid at this position, the prion conversion process could be promoted or inhibited [28].

### Generation and characterization of transgenic mice

**Fig 3:**
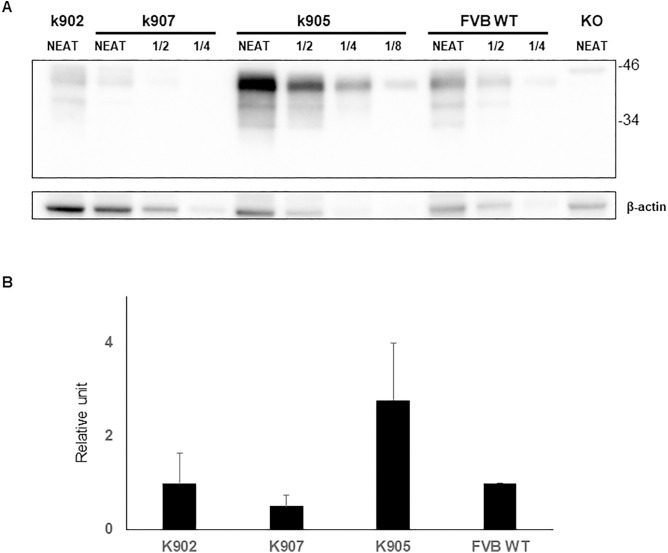
PrP^C^ expression levels of MoPrP H95Y in the different transgenic mice. A. Limit dilutions of brain homogenates of the mouse lines newly generated and wt FVB mice were subjected to WB in order to establish a comparison between the PrP^C^ expression levels among the different animals. k902 and k907 mouse lines expressed approximately the same level of wt FVB mice (1x for k902 and 0.5-1x for k907) whereas k905 expressed twice or four times as much endogenous wt PrP levels (2-4x). Molecular weight markers in kilodaltons (kDa) are provided. β-actin was used as a loading control. Immunoblot revealed with 12B2 Mab. B. Quantification of brain PrP^C^ levels normalized against β-actin. Four representative independent experiments were conducted and plotted in the diagram that displays the mean densitometric values. PrP^C^ levels in FVB WT brains were used as reference (1 relative unit). Error bars indicate the standard deviation.

The above mentioned results in cell lines encouraged us to generate transgenic mice expressing the H95Y mutation in the mouse *Prnp* gene. Three lines (founders) of MoPrP H95Y^+/-^ transgenic mice carrying the endogenous wt murine *Prnp* gene (MoPrP^+/+^ MoPrP H95Y^+/-^) were generated and named MoPrP^H95Y^-x902, MoPrP^H95Y^-x905 and MoPrP^H95Y^-x907 (from now just x902, x905 and x907 respectively). All lines were selected to be crossed with *Prnp* null mice (MoPrP^-/-^) to eliminate the endogenous wt murine *Prnp* gene and generate transgene-hemizygous lines (MoPrP^-/-^ Mo PrPH95Y^+/-^) which were named as MoPrP^H95Y^-k902, MoPrP^H95Y^-k905 and MoPrP^H95Y^-k907 (from now just k902, k905 and k907, respectively). Absence of the murine wt *Prnp* gene was determined using specific primers via PCR. Transgene expression levels on these last generated mouse lines were then determined in brain homogenates by serial dilution and densitometry and compared with PrP^C^ levels found in wt FVB mice brain homogenates. Transgene expression levels for the three different lines in 1-month-old mice were found to be ≈ 1x for k902, ≈ 0.5-1x for k907 and ≈2-4x for k905 respectively (Fig 3B). All lines showed an electrophoretic signature similar to that of the PrP^C^ present in wt FVB mice brain (Fig 3A).

### Spontaneous neurologic disease in transgenic mice expressing mutant MoPrP H95Y

A spontaneous neurologic disease was developed by the six different transgenic lines bearing the mutant MoPrP H95Y: (1) x902, (2) k902, (3) x907, (4) k907, (5) x905, and (6) k905 (Table 4). A complete monitoring of clinical signs of the founders and their descendants was carried out and mean survival times and attack rates were calculated on each case. Despite being both heterozygous with similar expression levels of PrP-WT and PrP-H95Y, Tg lines x902 and x907 (as well as k902 and k907) were analyzed as separate groups since they come from different founders. This allowed us to rule out that the effects eventually observed in these animals are due to genes affected by the integration site of the transgene.

**Table 4:** Spontaneous neurologic disease in transgenic mice expressing MoPrP H95Y.

Transgenic line	MoPrP H95Y expression level^a^	Endogenous wt-PrP^C^ expression level^a^	Onset of clinical signs, days ± SD^b^ (attack rate)^c^	Mean survival time ± SD^b^ (attack rate)^c^
x902	1x	1x	320 (1/6)	346^d^, > 600 (1/6)
k902	1x	0x	400 (1/6)	420^d^, > 600 (1/6)
x907	0.5-1x	1x	390 ± 60 (2/8)	443^d^; 467^d^, > 600 (2/8)
k907	0.5-1x	0x	400 (1/6)	450^d^, > 600 (1/6)
x905	2-4x	1x	97 ± 15 (9/9)	108 ± 19 (9/9)
k905	2-4x	0x	88 ± 10 (9/9)	100 ± 14 (9/9)

^a^One-month age animals compared to FVB wt mice brain.

^b^Standard deviation.

^c^Proportion of PrP^res^-positive mice among all the mice inoculated.

^d^Age of death of PrP^res^-positive animals.

The development of the disease and the onset of clinical signs and survival times were strongly dependent on the expression level of mutant MoPrP H95Y. Transgenic lines x902, k902, x907 and k907 expressing reduced levels of the mutant protein displayed low attack rates (1 or 2 PrP^res^ positive animals out of 6 or 7 total animals) with long survival from 346 to 467 days of age and late onset of clinical signs: 320 days for x902 line, 400 days for k902 line, 390 ± 60 days for line x907 and 400 days for k907. Whereas transgenic lines x905 and k905, which express double/quadruple levels of the mutant protein than the other transgenic lines, displayed a disease fully penetrating and with a fast development: 100% attack rates with early onset of the clinical signs (97 ± 15 and 88 ± 10 days for x905 and k905 respectively) and an extremely reduced lifespan of 108 ± 19 and 100 ± 14 days of age for x905 and k905 lines, respectively. It is important to note that the time to death once the clinical signs appeared was extremely short, around 10 days.

Clinical signs generally involved motor impairment in combination with pronounced ataxia. Other observed signs were rough coat, prominent hunch and wobbling gait. At the end stage of the disease, paralysis in the back limbs was usually observed. No signs of hyperactivity or lethargy were detected in any of the lines.

### Brain PrP^res^ associated to the spontaneous neurologic disease in transgenic mice expressing mutant MoPrP H95Y

**Fig 4:**
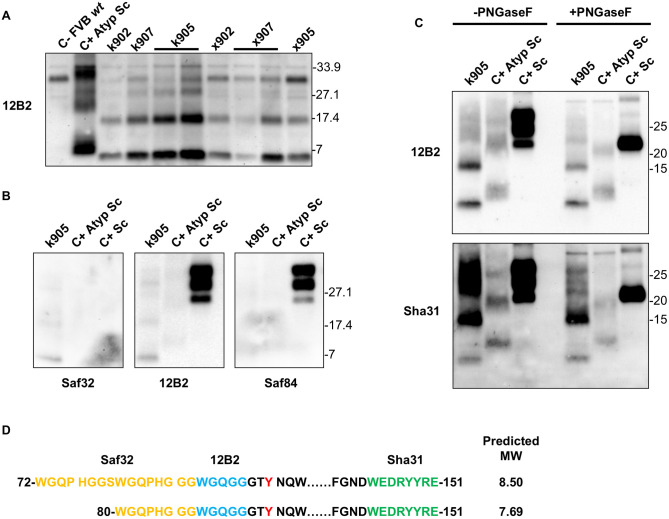
Characterization of the brain PrP^res^ present in the different mouse lines generated in this study. A. WB showing the biochemical signature of the PrP^res^ accumulated in the brains of the different wt mice (negative control, first lane on the left: wt FVB mice uninoculated; positive control, second lane on the left: atypical scrapie sample Nor98; and MoPrP H95Y mouse lines (as listed) as revealed with the Mab 12B2. All the animals exhibited a similar atypical PrP^res^. Molecular weights in kDa are shown on the right of the panel. Onset of disease is described in Table 4. B. Epitope mapping of the brain PrP^res^ from k905 mice probed with Saf32, 12B2 and Saf84. As positive control a classical scrapie prion (C + Sc) and an atypical scrapie Nor98 (C+ Atyp Sc) are included. Molecular weights in kDa are shown on both sides of the panel. C. Degycosylation of the PrP^res^ of k905 mice. The two characteristic small fragments of 17kDa and below 7kDa are detected after deglycosylation, as well as an upper band above 21kDa. As positive control, a classical scrapie prion (C + Sc) and an atypical scrapie Nor98 (C+ Atyp Sc) are included. Deglycosylation treatment with PNGaseF is marked as +PNGaseF. Molecular weights in kDa are shown on the right of the panel. D. Predicted molecular mass in kDa of the PrP^res^ from MoPrP H95Y mice based on the epitope mapping detection using ExPASy software. Amino acid numbers refer to mouse sequence and the mutation of the H to Y in position 95 is marked in red. Epitopes of the antibodies used for characterization are highlighted in colors: orange for Saf32, blue for 12B2 and green for Sha31.

All mice were sacrificed due to ethical reasons in the presence of clinical signs or at the end of their lifespan and their brain was analyzed for the presence of PrP^res^. The signature PrP^res^ present in the brains of diseased animals was similar to the one of Nor98 atypical scrapie with a band of ≈17–18 kDa and a main band of ≈7–8 kDa [29] and identical between the different mouse lines expressing the mutated MoPrP H95Y (Fig 4A and 4B). Mainly, brain PrP^res^ in these mice was characterized by two small PK-resistant fragments, one of approximately 17 kDa and a smaller one below 7 kDa.

Deglycosylation of the k905 PrP^res^ with PNGase F showed these two bands as well as an upper band above 21 kDa (Fig 4C). The glycotype of MoPrP H95Y PrP^res^ was detected both by 12B2 and Sha31 Mab without major differences between these antibodies. Therefore, an epitope mapping with more N-terminal and C-terminal PK cleavage site antibodies was performed (Fig 4B). Interestingly, the characteristic PrP^res^ fragments of the MoPrP H95Y were recognized with Saf32, whose epitope is within the octarepeat region. This antibody did not recognize the atypical scrapie Nor98 PrP^res^, a result in line with other previous studies that discussed an interference due to the G92 insertion [30]. The C-terminal antibody Saf84 did not detect any of the PrP^res^ fragments. Based on these epitope observations, a predicted molecular mass of 7.69-8.5 kDa was estimated using ExPASy software (Fig 4D). The predicted fragment is compatible with the small PrP^res^ below 7kDa detected by WB. Considering that all antibodies tested showed the same recognition pattern and the deglycosylation experiments, the ≈ 17kDa band could consist of a dimer of the lower fragment (as hypothesized for atypical scrapie Nor98 [31]). Additionally, the kinetics of PrP^Sc^ resistance to PK (S3 Fig) showed a sensitivity of the brain PrP^res^ from MoPrP H95Y similar to atypical scrapie Nor98 prion.

**Fig 5:**
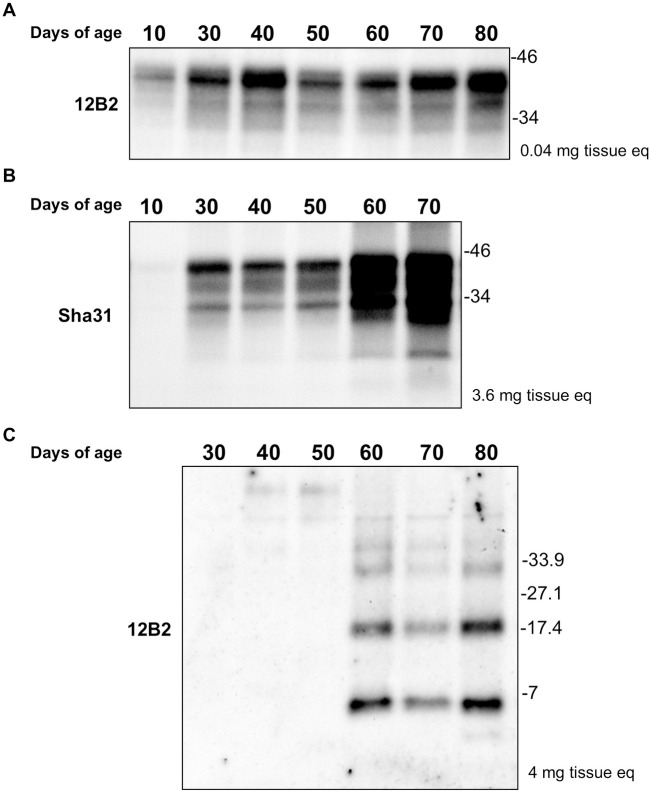
Study of the stability of PrP^C^ expression levels, insoluble PrP^Sc^ and PrP^res^ accumulation in k905 transgenic line. A. Immunoblotting revealed with the Mab 12B2 of the stability of PrP^C^ expression levels into k905 animals of different ages. PrPC level was stable beyond 30 days of age. B. Immunoblotting revealed with Mab Sha31 of insoluble PrP^Sc^ accumulation in k905 mice of different ages. Beyond 30 days of age the insoluble PrP^Sc^ was detected. After 60 days, the amount of insoluble PrP^Sc^ increased and became stable (non-linear progression). C. Immunoblotting as revealed with the Mab 12B2 of PrP^res^ accumulation into k905 animals of different ages. Brain PrP^res^ was detected only after 60 days of age, therefore, increasing over time with a non-linear progression. Molecular weight markers in kDa are provided on the right hand-side of the figure. The amount of loaded sample measured in mg of brain equivalent is indicated in the right side of the blot (mg tissue eq).

The stability of PrP^C^ levels and the progressive accumulation of PrP^res^ during the whole life were then studied in the k905 transgenic line (Fig 5). In this transgenic mouse line, PrP^C^ levels seem to be stable in time beyond 30 days of age (Fig 5A). After 30 days, an insoluble PrP^Sc^ was detected, and after 60 days, the accumulation of this form increased and became stable (Fig 5B). However, brain PrP^res^ was only detectable in animals after 60 days of age, approximately 20 days before the onset of clinical signs and 30 days before the animals’ death (Fig 5C).

### Transmission *of* neurodegenerative disease *from* transgenic mice overexpressing MoPrP H95Y *to* other mice

To test the potential infectivity of diseased MoPrP H95Y transgenic mice, inocula were prepared from PrP^res^ positive brains of x905, k905 and x907 individual (see Table 5 for further details). k907, *Tga20* and FVB mice were intracranially challenged with these inocula. The k907 transgenic mouse line was chosen due to its low rate of development of the spontaneous disease. Moreover, only K907, and not k902, was selected for inoculation because no differences were found between the two lines that would justify the duplication of the experiments and the use of a high number of animals.

**Table 5:** Transmission analysis of brain homogenate from spontaneously diseased mouse lines.

Inoculum	Mean survival time ± SD^a^ (attack rate)^b^		
	Mouse line		
	k907	*Tga20*	FVB (wt mice)
x905^c^	224 ± 51 (7/7)	173 ± 8 (5/5)	>600 (0/5)
k905^c^	244 ± 28 (6/6)	144 ± 11 (4/4)	>600 (0/8)
x907^c^	299 ± 61 (7/7)	178 ± 10 (6/6)	>600 (0/5)
x905/FVB^d^	ND^e^	192 ± 14 (6/6)	>600 (0/5)
None	450^f^, > 600 (1/6)	>600 (0/6)	>600 (0/6)

^a^Standard deviation.

^b^Proportion of PrP^res^-positive mice among all the inoculated mice.

^c^Pool of several brains from animals affected by the spontaneous pathology.

^d^Pool of several brains from FVB mice inoculated with x905 inoculum.

^e^Not done.

^f^Age of death of the PrP^res^-positive animal.

The *Tga20* transgenic line was chosen because of its high expression levels of wt mouse PrP.

**Fig 6:**
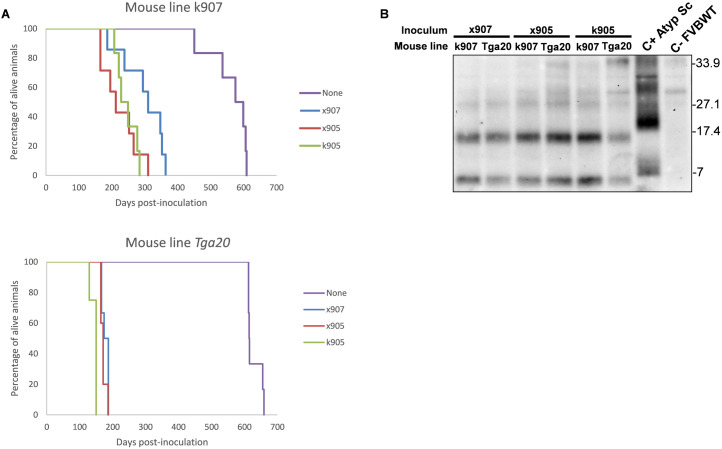
Transmission of spontaneous disease to k907 mouse line and Tga20 transgenic mice expressing wt MoPrP. A. Survival curves for the different mouse lines. The incubation times were shorter in the Tga20 line possibly due to its higher expression of MoPrP. B. Immunoblotting as revealed with the Mab 12B2 showing that the PrP^res^ signature of the original inoculum is maintained after passage into both k907 and Tga20 mouse lines. Atypical scrapie Nor98 was included as positive control, second lane on the right (C+ Atyp Sc) and wt FVB mice uninoculated as negative control, first lane on the right (C- FVB wt). Molecular weight markers in kDa are provided.

Transmission of x905, k905 and x907 inocula was achieved with 100% attack rates and short incubation times in the groups of animals from k907 and *Tga20* lines, proving the transmissibility of the spontaneous disease associated to H95Y mutation (Table 5). The survival times were different between k907 and *Tga20* inoculated animals, being shorter in the *Tga20* transgenic line possibly due to its higher expression levels of mouse PrP (Table 5 and Fig 6A). There was neither brain-PrP^res^ nor disease detection upon transmission to the FVB mice on the first and second passage. However, the transmission of a pool of brains from FVB mice inoculated with x905 inoculum to the *Tga20* mice was achieved with a 100% attack rate and low incubation times. The results in k907 can be interpreted simply as a case of seeding that accelerated the proper spontaneous disease in k907 animals. However, the low attack rate of the spontaneous disease on these mice and its long incubation times are clearly opposite to the 100% attack rate and short incubation times observed in k907 injected animals. Thus, the result suggests that inoculated k907 died because of the transmission of the prion disease and not due to prion seeding.

From a biochemical point of view, regardless of the inoculum, the PrP^res^ signature in the k907 and *Tga20* inoculated animals is the same of the one of the original inoculum, an atypical PrP^res^ characterized by two small PK-resistant fragments, one of approximately 17 kDa and other smaller below 7 kDa (Fig 6B).

### Neuropathological alterations *in* transgenic mice expressing mutant MoPrP H95Y

**Fig 7:**
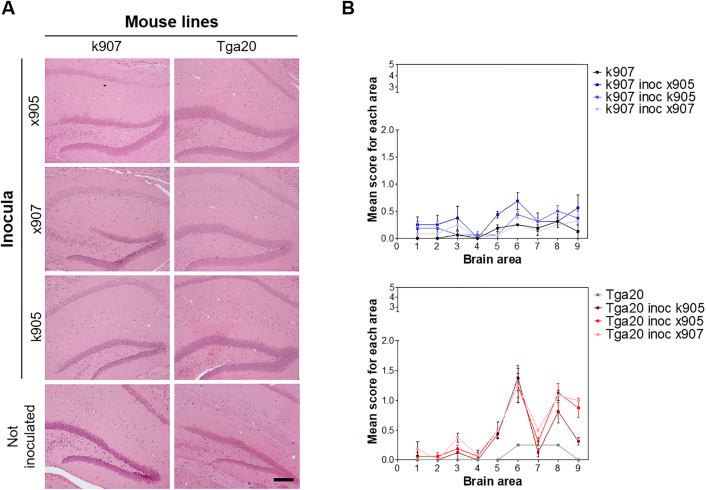
Neuropathological characteristics of MoPrP H95Y mouse lines. A. Representative Hematoxylin and Eosin images of the hippocampus. Scale bar: 40 µm. B. Lesion profiles showing higher spongiosis in Tga20 inoculated mice, compared to k907. Vacuolation was scored in nine gray matter areas on a scale of 1-5: 1, dorsal medulla; 2, cerebellar cortex; 3, superior colliculus; 4, hypothalamus; 5, thalamus; 6, hippocampus; 7, septum; 8, retrosplenial and adjacent motor cortex; 9, cingulate and adjacent motor cortex.

The histological examination of mice showed no spongiform changes in uninjected k907 and *Tga20* mice. Moderate spongiosis, primarily localized in the hippocampus and frontal cortex, was observed in K907 mice inoculated with all brain homogenates (Fig 7A). Slightly more pronounced alterations were observed in the brains of *Tga20* animals challenged with the same inocula. Again, the hippocampus and frontal cortex were the most affected regions (Fig 7).

**Fig 8:**
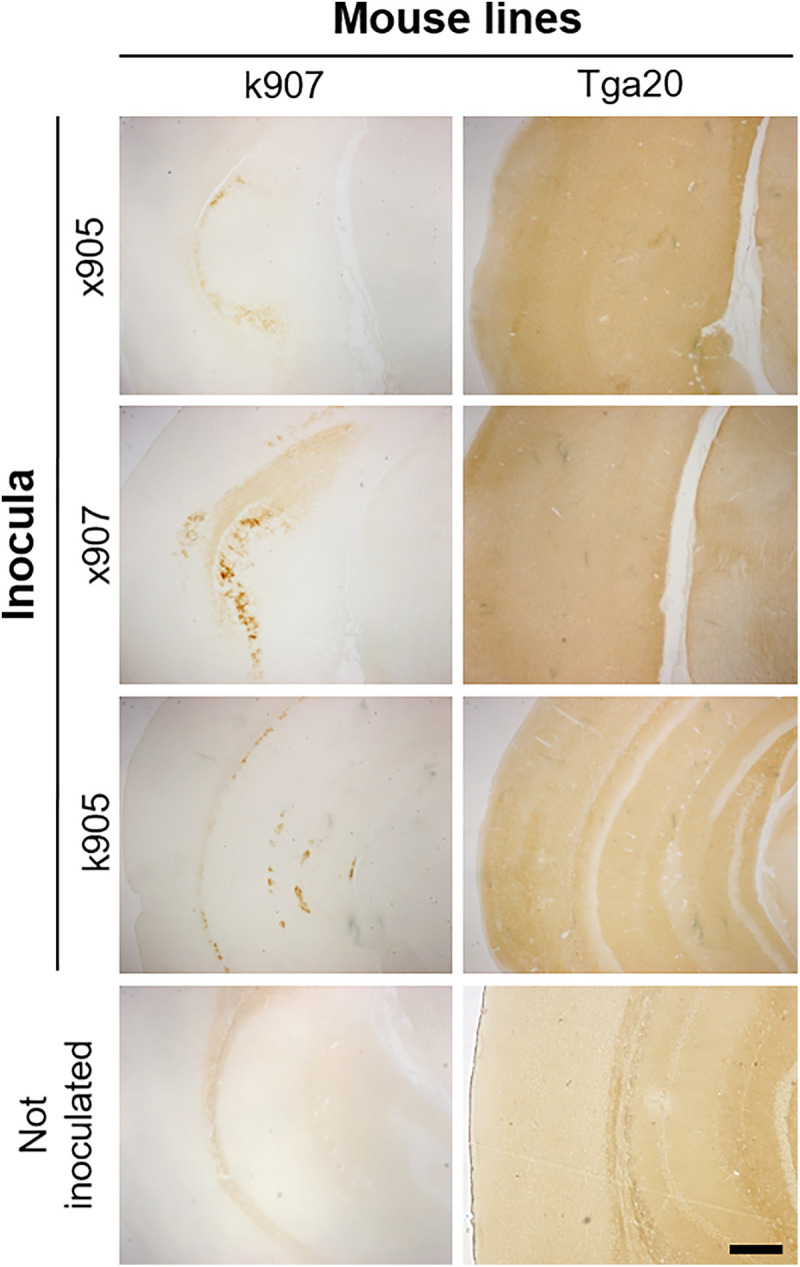
Detection of PrP^res^ in injected mice. Sections were digested with PK 5 µg/mL and immunostained with EB8 antibody. K907 injected mice showed the presence of PrP^res^ in the subiculum. Tga20 injected mice still showed the presence of PrP^C^, not completely digested by PK, due to the overexpression of the protein. Scale bar: 40 µm.

The atypical PrP^res^ observed in WB showed greater sensitivity to PK compared to the traditional prions. Thus, for the immunohistochemical analyses, the samples were treated with a lower concentration of PK (5µg/mL), and this allowed us to observe a PK-resistant signal in the subiculum of all k907-inoculated mice but not in the same uninoculated animals (Fig 8). Unfortunately, the low concentration of PK used was insufficient to digest all the PrP^C^ present in *Tga20* mice, thus hampering the possibility to identify the atypical prion in the brain of these mice. In contrast to Saf-61 and 6H4, only the EB8 antibody, which recognizes the N-terminal region, allowed for PrP^res^ detection (S4 Fig). Except for the subiculum, we did not detect PrP^res^ deposition in any other region of the brain. This may be attributed to the fact that several portions of the atypical PrP^res^ generated in these animals may have been lost after PK digestion. The use of a higher concentration of PK hindered the possibility to detect PrP^res^ signal in the subiculum of k907 mice, while the use of lower concentrations did not adequately digest the PrP^C^ in both animal groups.

In spite of the difficulties in detecting PrP^res^ in the brains of these animals, GFAP activation was observed in all injected mice primarily affecting the hippocampus, frontal cortex and thalamus.

**Fig 9:**
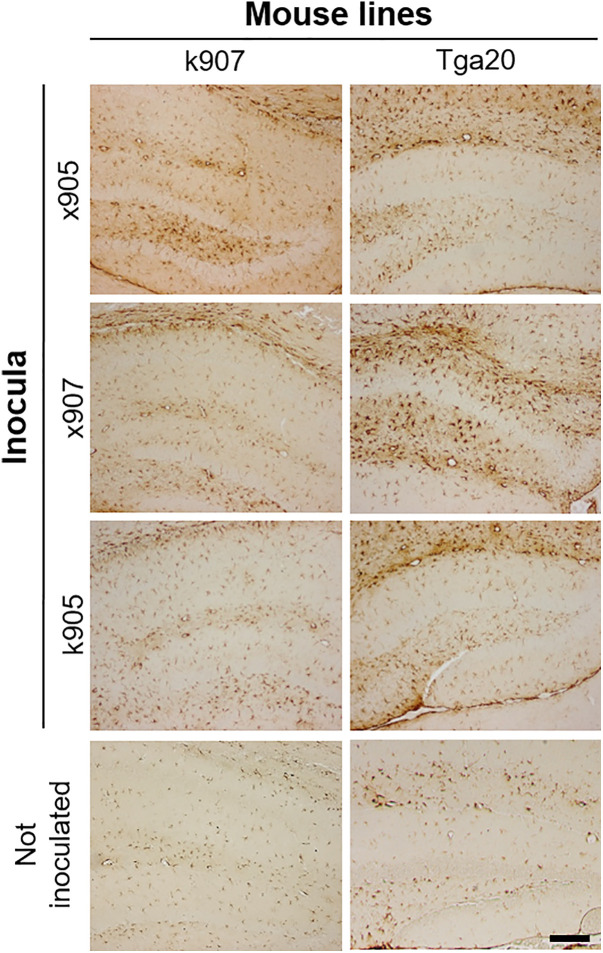
GFAP activation in the brain of k907 and Tga20 mice. All of the injected animals showed a marked astroglial activation. Scale bar: 40 µm.

Interestingly, the activation was more severe in the brain of *Tga20* challenged mice (Fig 9).

## Discussion

In this study, we identified an amino acid substitution of a single residue in the prion protein that is able to promote spontaneous prion disease in mice. This mutation alters the propensity of the protein to coordinate copper ions in a region in the N-terminal, denoted as the non-OR copper binding site. The prion protein has been known for a long time as a polypeptide able to bind copper ions [32,33]. Many studies have addressed the role of copper in the folding and misfolding of the prion protein [5]. The copper coordination architecture in the N-terminal domain of the prion protein has been resolved and many sequential modes of binding have been proposed [34,35]. These studies were paralleled with those aiming at defining physiological roles for copper coordination in the prion protein. Copper coordination seems to modulate its several physiological functions: from neuritogenesis [36,37] to N-Methyl-d-aspartate (NMDA) receptors regulation [38]. On the other hand, the role of copper coordination in prion formation has been quite controversial: whether copper presence or the lack of it in the prion protein is important for its infectivity remains to be established [7,39]. Nevertheless, strong evidence for prion formation in modulating NMDA receptors has been recently provided [40,41]. Indeed, prion conversion and accumulation act through copper modulation of S-nitrosylation of NMDA receptors [38,40].

Following early work on copper coordination and structure, we were motivated to explore the conformation of the prion protein in its functions and in the presence of point mutations [11]. We noticed that pathological mutations present in the C-terminus, like most mutations in the prion protein, affected copper coordination in the non-OR and in position H95 [9,12,42]. We therefore decided to modify copper coordination in the various residues of the N-terminus of the prion protein to verify whether this change would affect prion formation or infectivity. Earlier work has indicated that changes of the histidine residues may affect the propensity of replicated prions. In fact, a mutagenesis study in which each histidine at the N-terminus was substituted by glycine residues evaluated the susceptibility to prion inoculation in transgenic mice. The substitution of all histidines at the OR region did prolong the incubation time upon inoculation with prions, a substitution at position H95 accelerated the disease [10].

While these observations support a potential role of copper in modulating prion conversion, our data may also point to a copper-independent mechanism driven by changes in the folding environment of the cellular prion protein. In fact, histidine 95 is located adjacent to a conserved hydrophobic palindromic domain, shown implicated in the early stages of PrP^C^ misfolding [43]. Because of the close proximity, the histidine residue substitution might directly affect the structural environment of the hydrophobic domain. As demonstrated, indeed, hydrophobic replacements, such as tyrosine, enhance prion formation, possibly by stabilizing hydrophobic interactions that favor β-sheet assembly within the PrP^Sc^ conformation; in contrast, substitution with electrically charged residues impairs conversion, probably due to electrostatic disruption of this local structural disposition (data presented in Fig 2). Notably, transgenic studies have shown that PrP variants lacking the octarepeat region but maintaining the non-octarepeat segment can still favor prion propagation, while deletion of the entire N-terminal domain up to residue 121 completely abolishes prion conversion [44]. These findings point toward an important contribution of the non-OR domain, not only in coordinating metal ions, but also in supporting the folding configurations and the interactions between residues necessary for PrP^C^ to convert into its misfolded isoform.

Finally, we should consider another mechanism that has been recently proposed. Not only the histidines at N-terminus may contribute to the correct folding of PrP but also the histidines present at the C-terminus. Whether or not the folding of the N-terminus back to the C-terminus has a role, mutation in the residues involved in copper coordination may affect the ‘close’ conformation to advantage the ‘open’ one [45].

In our work, we have defined that H95 is the most important residue present in the N-terminus involved in copper coordination. Changes of H95 with any other residue modulate/modify the propensity of the protein in becoming infectious. We decided to study the H95Y mutation since Y occupies a similar geometrical space as H. There is a single reference on a human pathological mutation leading to prion disease, reported in an individual carrying the homologous H96Y [46]. Unfortunately, no additional information is available besides the genetic screening and the post-portem confirmation that, indeed, it was a prion disease [46]. We have previously analyzed the role of H95 in prion formation in cultured cells [12]. In this report, we recapitulate our studies on H95Y in cells. We show that each substitution of the histidine residues present at the OR region at N-terminal moiety did not affect prion formation, while we show biological evidence for the role of H95 in prion formation. In particular, H95Y enhances the accumulation of prions in cell cultures.

We therefore decided to create transgenic mice bearing the PrP mutation H95Y.

In this work, by establishing a series of transgenic mouse models, we describe that indeed H95Y leads to spontaneous prion disease in transgenic mice. There are several features that are unique about these models.

First, the glycotype biochemical pattern of PK-resistant PrP. The biochemical characterization of PrP^res^ resembles the one of Nor98, a sheep sporadic disorder [29]. As for Nor98, H95Y possesses a unique PK-digestion pattern. The glycotype is different from other known prion strains and as such it may indicate that the prion conformation is dissimilar from classical prion strains patterning. Second, the spontaneously generated prion is transmissible. The disease is accelerated in recipient mice bearing the same mutation but not in wild-type animals. Several attempts to establishing disease in wild-type animals failed mostly because of the transmission barrier phenomenon. This is quite common in prion disease research since the age of wild-type mouse models may not sustain longer incubation times that the actual age of the animals. In this study, the transmission barrier was overcome by over-expression of the wild-type prion protein molecule in transgenic mice (*Tga20* mice). Third, the neuropathology of the inoculated animals followed the unicity of this newly generated prion strain. Indeed, identifying prion deposits through immunohistochemistry proved challenging. When deposits were detected using low concentrations of PK, they localized to a specific region of the brain. Particularly, the k907-inoculated animals with various brain homogenates showed spongiform alterations and the presence of an atypical prion in the subiculum, which was not observed in the brains of the same line of uninoculated animals. The same inocula were also able to induce more pronounced spongiform alterations in *Tga20* mice; however, in these mice we could not demonstrate the presence of prions through IHC due to the insufficient concentration of PK used to completely digest the overexpressed PrP^C^ in this mouse line. In both mouse lines (k907 and *Tga20*) challenged with brain homogenates, we observed marked astroglial activation, suggesting the presence of ongoing pathological processes likely associated to atypical prions propagation.

Spontaneous prion disease in transgenic mice has also been described in transgenic mouse models harboring mutations in the non-OR region of the PrP sequence. For example, transgenic mice Tg(A116V) [47] or 117VTg30 [48] with the A117V mutation in murine and human PrP sequence respectively, the Tg(MoPrP-P101L) [49] and 113LBoPrPTg [50] carrying the P102L mutation in the murine and bovine PrP or the TgShI112 [51] mice that incorporate the M112I mutation in the sheep sequence (homologous to the M109I in mouse sequence), all exhibit this phenomenon. Interestingly, these models also displayed spontaneous disease features similar to Nor98 atypical prions, particularly PrP^res^ signature and histopathological analysis. These similarities with the transgenic mice with the H95Y mutation suggest that these models may follow a common pathological process.

In addition, it is now widely accepted that bank vole PrP is a universal acceptor for prions and indeed the sequence homology points out at the role of residue 108, methionine in mouse and isoleucine in bank vole. This residue lies within the non-OR sequence, just before the second histidine that coordinates copper [52].

Therefore, we could hypothesize that the alteration of the non-OR architecture would lead to spontaneous misfolding via the alteration of the non-OR histidines copper cooperation role.

In conclusion, we describe a novel spontaneous prion disease in mice due to a single substitution of a histidine in position 95 to a tyrosine. This mutation, together with previous work from our laboratory, establishes the importance of copper coordination in the non-OR region of the prion protein as a major determinant for prion conversion and replication.

## Supporting information

S1 Fig The 3F4–epitope tag has no effect on prion conversion. Transiently transfected ScN2a cells with vector pcDNA-MoPrPWT or pcDNA-MoPrP3F4WT, to confirm that the tag has no effect on prion replication. For non-PK experiment, fifty μg of undigested lysates was applied to each lane, β-actin was used as internal control. For PK experiment, five hundred μg of cell lysates was digested with PK (20 μg/mL) at 37°C for 1 hour. PrPs were detected by anti-PrP W226 mAb (A) or 3F4 mAb (B). Molecular weight markers in kilodaltons (kDa) are provided.
(TIF)

S2 Fig The histidine substitutions in the OR and non-OR regions are not toxic for cell culture. MTT assay performed 72 hours post transfection, showing that expressions of WT3F4 and mutant MoPrP constructs had no toxicity effect on ScN2a cells.
(TIF)

S3 Fig Titration of proteinase K digestion of the brain PrP^res^ from MoPrP H95Y mice. Immunoblotting of brain PrP^res^ from k905 mice and an atypical scrapie Nor98 control (C+ Atyp Sc) digested with a range of increasing concentration of PK (from 0 to 120 μg/ml), revealed with 12B2 Mab. Dilution factor of the loaded samples is provided. Molecular weight markers in kilodaltons (kDa) are included on the left side of the blots.
(TIF)

S4 Fig Immunohistochemical analysis of PrP^res^ using different antibodies. Representative brain sections from transgenic mice 195Y and 195H, inoculated and not inoculated, were stained with anti-PrP antibodies: Saf-61 (A) and 6H4 (B). No PrP^res^ signal was detected in any of the experimental groups, whereas a clear positive signal was observed in RML-infected CD1 mice used as positive control.
(TIF)
