# Supplementary figures and images for: Substitution of histidine 95 by tyrosine in the prion protein causes spontaneous neurodegeneration in transgenic mice
Source: PLoS Pathog. 2025 Oct 16;21(10):e1013554. doi: 10.1371/journal.ppat.1013554 (PMC12530561; doi:10.1371/journal.ppat.1013554)

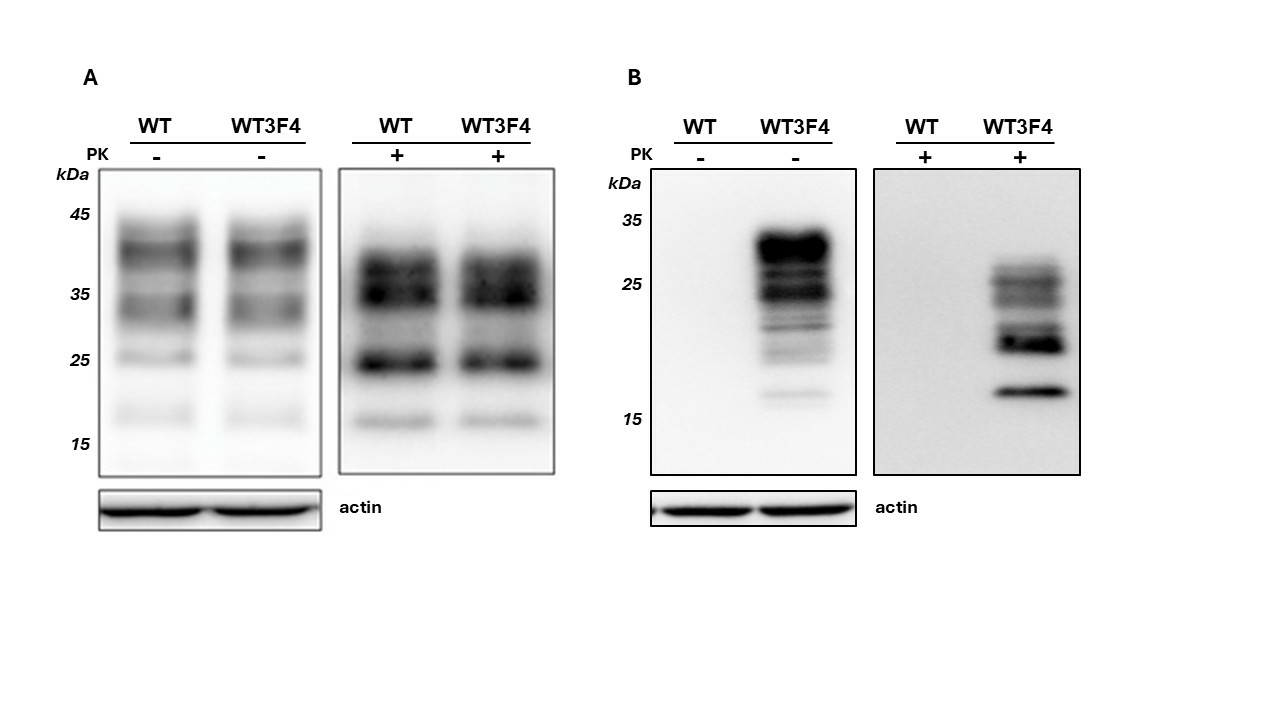

Supplement: S1 Fig — Transiently transfected ScN2a cells with vector pcDNA-MoPrPWT or pcDNA-MoPrP3F4WT, to confirm that the tag has no effect on prion replication. For non-PK experiment, fifty μg of undigested lysates was applied to each lane, β-actin was used as internal control. For PK experiment, five hundred μg of cell lysates was digested with PK (20 μg/mL) at 37°C for 1 hour. PrPs were detected by anti-PrP W226 mAb (A) or 3F4 mAb (B). Molecular weight markers in kilodaltons (kDa) are provided. (TIF) [file ppat.1013554.s001.tif]

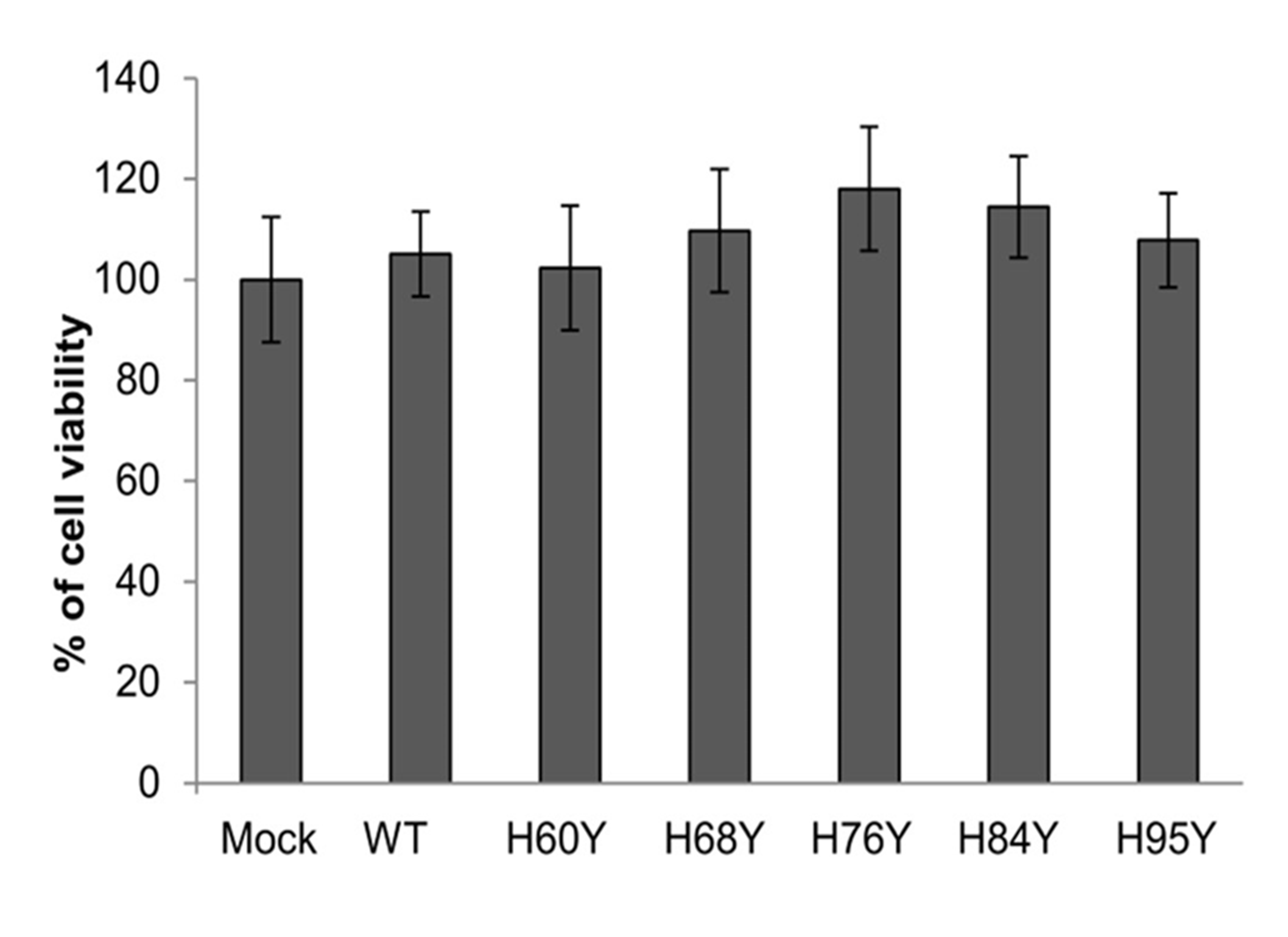

Supplement: S2 Fig — MTT assay performed 72 hours post transfection, showing that expressions of WT3F4 and mutant MoPrP constructs had no toxicity effect on ScN2a cells. (TIF) [file ppat.1013554.s002.tif]

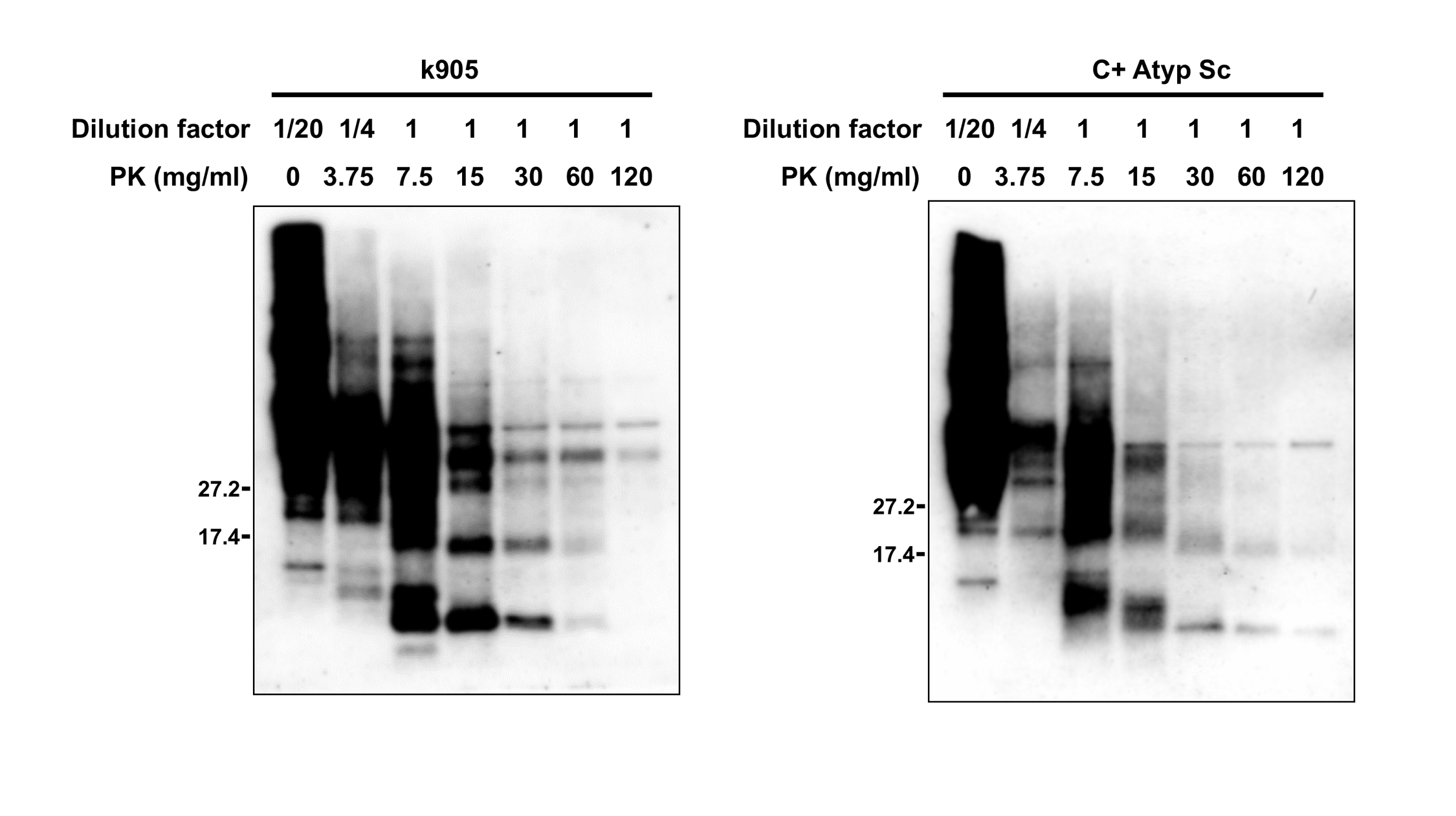

Supplement: S3 Fig — Immunoblotting of brain PrPres from k905 mice and an atypical scrapie Nor98 control (C+ Atyp Sc) digested with a range of increasing concentration of PK (from 0 to 120 μg/ml), revealed with 12B2 Mab. Dilution factor of the loaded samples is provided. Molecular weight markers in kilodaltons (kDa) are included on the left side of the blots. (TIF) [file ppat.1013554.s003.tif]

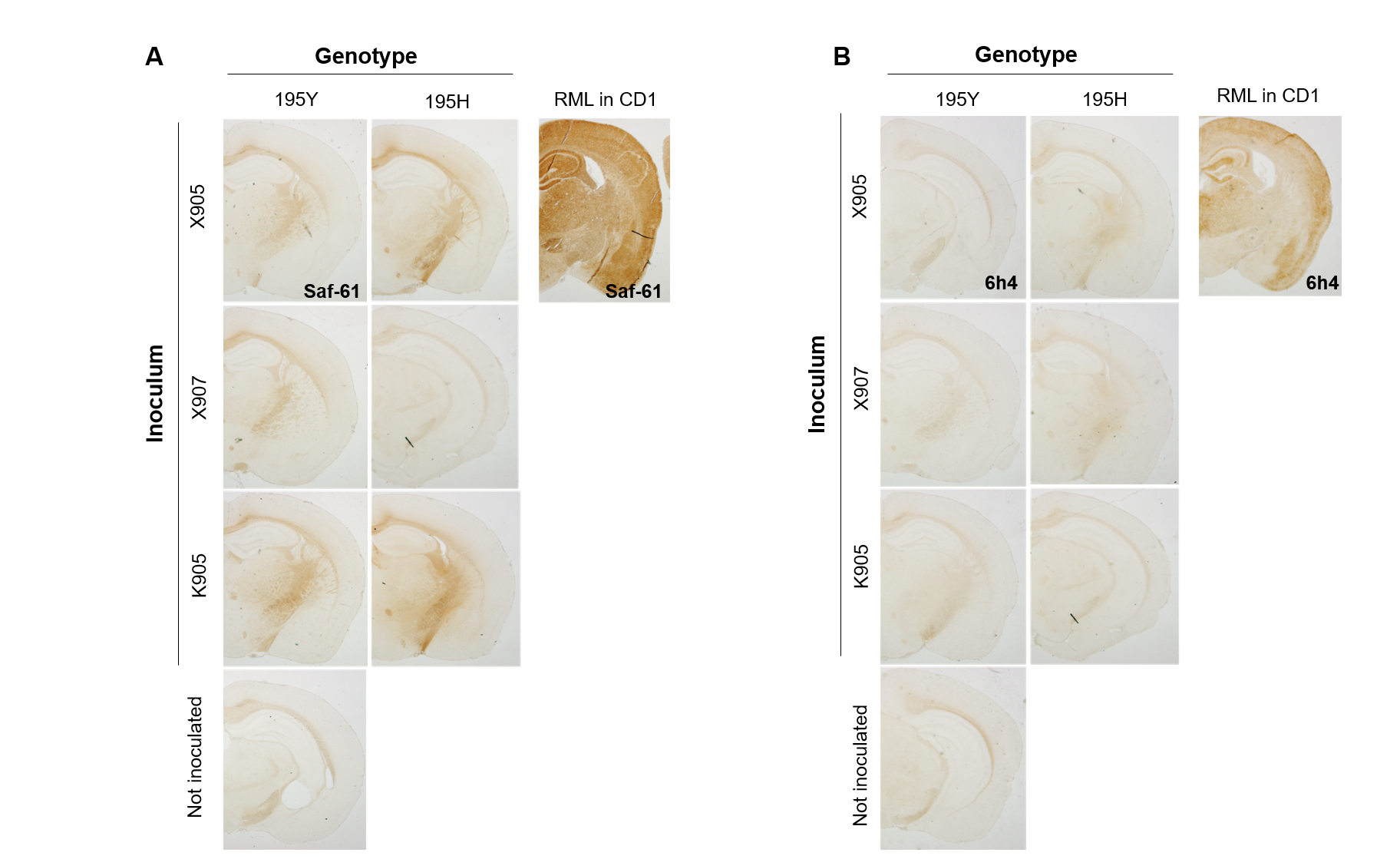

Supplement: S4 Fig — Representative brain sections from transgenic mice 195Y and 195H, inoculated and not inoculated, were stained with anti-PrP antibodies: Saf-61 (A) and 6H4 (B). No PrPres signal was detected in any of the experimental groups, whereas a clear positive signal was observed in RML-infected CD1 mice used as positive control. (TIF) [file ppat.1013554.s004.tif]
